# Bifunctional Enzyme JMJD6 Contributes to Multiple Disease Pathogenesis: New Twist on the Old Story

**DOI:** 10.3390/biom7020041

**Published:** 2017-06-01

**Authors:** Shiva Shankar Vangimalla, Murali Ganesan, Kusum K. Kharbanda, Natalia A. Osna

**Affiliations:** 1 Research Service, Veterans Affairs Nebraska-Western Iowa Health Care System, 4101 Woolworth Avenue, Omaha, NE 68105, USA; Shankar.vang@gmail.com (S.S.V.); murali.ganesan@unmc.edu (M.G.); kkharbanda@unmc.edu (K.K.K.); 2 Department of Internal Medicine, University of Nebraska Medical Center, Omaha, NE 68198, USA

**Keywords:** JMJD6, methylation, cancer, viruses

## Abstract

Jumonji domain-containing protein 6 (JMJD6) is a non-heme Fe(II) 2-oxoglutarate (2OG)-dependent oxygenase with arginine demethylase and lysyl hydroxylase activities. Its initial discovery as a dispensable phosphatidylserine receptor (PSR) in the cell membrane of macrophages for phagocytosis was squashed by newer studies which revealed its nuclear localization and bifunctional enzymatic activity. Though its interaction with several nuclear and cytoplasmic target proteins has been demonstrated, the exact mechanisms and clinical significance of these various biologic interplays are not yet well established. Recent investigations have shed the light on the multiple pathways by which JMJD6 can regulate cell proliferation and cause tumorigenesis. Clinically, JMJD6 has been associated with more aggressive and metastatic disease, poorer prognosis, and lower overall survival rates—particularly in lung colon and oral cancers. JMJD6 is a novel biomarker for predicting future disease outcomes and is a target for new therapeutic treatments in future studies. Aberrant expression and dysregulation of JMJD6 are implicated in various other processes such as impaired T-cell proliferation and maturation, inoculation, and virulence of foot-and-mouth disease virus (FMDV), and impaired methylation of innate immunity factor. This article reviews the association of JMJD6 with various pathological processes—particularly, its role in tumorigenesis and virological interactions.

## 1. Introduction

In the early 1960s, protein methylation was first described with the discovery of *ε*-*N*-methyl-lysine in the flagellar protein of *Salmonella typhimurium* [1]. Later, lysine residues of histones were found to be methylated [2]. Over the years, an increasing number of protein methylation reactions were discovered by various groups [3,4,5]. It is now a well-documented phenomenon that methylation is one of the observed common forms of post-translational modifications. Similar to other types of post-translational modifications, protein methylation has been implicated in a plethora of cellular effects such as regulation of transcription [6], signal transduction [7], protein trafficking, protein repair [8], T-cell activation [9,10], nuclear transport [11], etc. Depending on the type of methylation, the protein–protein interactions can be either promoted or inhibited, thus expanding the repertoire of chemistry that a protein can perform [12].

*S*-Adenosyl Methionine (SAM) serves as a major methyl donor for many of the transmethylation reactions. These reactions are, in turn, catalyzed by a diverse set of methyltransferases resulting in the production of methylated biomolecules such as phospholipids, proteins, nucleic acids, and other small molecules [13,14]. It is estimated that over 1% of genes in the mammalian genome encode for these methyltransferases [15]. Modulation of methyltransferase activity through SAM:SAH (*S*-adenosyl homocysteine) ratio directly regulates protein methylation. However, the protein levels and functions can be regulated by histone or DNA/RNA methylation. Histone methyltransferases catalyze the transfer of methyl groups to arginine and lysine residues of histone proteins [16,17], while DNA/RNA methyltransferases primarily methylate the 5-carbon of the base cytosine of DNA [18]. Both types of methyltransferases were extensively investigated because of their direct and indirect role in epigenetic modification of chromatin. This modification, in turn, determines gene expression, genomic stability, cell differentiation, embryonic development, etc. The aberrant expression and enzymatic dysregulation of these methyltransferases have been reported in association with several diseases, including many types of cancer [19].

The methylation story has continued to grow exponentially with new developments over the last decade of the 20th century. This has also raised controversy over the reversibility of methylation as no demethylases had been identified at that time. Around 2004, the discovery of deiminase enzymes—which convert unmodified and mono-methylated arginine residues into citrulline residues by demethylimination reactions—laid to rest the controversy surrounding the reversibility of methylation reactions [20,21]. However, these enzymes were not interpreted as true demethylases as they produced citrulline residues in the absence of unmodified arginine residues. The first arginine demethylase producing unmodified demethylated arginine residues, namely Jumonji domain-containing protein 6 (JMJD6), was reported in 2007 [22]. There are still debates whether JMJD6 is a true arginine demethylase as recently, it has been suggested that acting as JMJC histone lysine demethylase, JMJD6 catalyzes arginine demethylation rather than possesses the direct arginine demethylating activity [23]. In this review, we summarize the basic concepts about JMJD6 emphasizing the history, structure, function, and a few known clinical associations—particularly, its role in tumorigenesis and virological interactions.

## 2. History of JMJD6

In 2000, JMJD6 was discovered as a phosphatidylserine receptor (PSR) on the cell membrane of macrophages with a possible role in the phagocytosis of apoptotic cells [24]. However, in 2004 Cikala et al., identified the distorted double-stranded B-helix (DSBH) fold along with possible nuclear localization signals (NLS) of the protein [25]. It was suggested that it is a 2-oxoglutarate (2OG)-dependent dioxygenase localizing to the nucleus based on the similarities between predicted JMJD6 structure and other 2OG-dependent oxygenase enzymes, such as hypoxia-inducible factor (HIF)-alpha asparaginyl hydroxylase FIH (factor inhibiting HIF) [25]. Due to this prediction, it was named as JMJD6. Around the same time, in vivo experiments with JMJD6 knockout mice (PSR knockout mice) revealed the death of mice at the time of birth with severe embryonic defects, including malformations of the brain, eye defects, lung defects, impaired cell differentiation, kidney defects, heart defects, etc. [26,27]. Experiments in zebrafish involving morpholinos directed against JMJD6 yielded similar results [28].

In 2007, for the first time, the arginine demethylase activity of JMJD6 on histone proteins was experimentally characterized by Chang et al. [22]. In 2009, Webby et al., demonstrated the lysine hydroxylase activity of JMJD6 on splicing associated factors, such as U2AF65 [29]. Both of these findings gave rise to the theory of bifunctional enzymatic activity of JMJD6. However, while over the years many studies corroborated the lysine hydroxylase activity of JMJD6, the same cannot be attributed to its arginine demethylase activity. The demethylase activity of JMJD6 has been challenged over the years by many other independent study groups who reported the absence of *N*-methyl arginine demethylation activity of JMJD6 in their histone protein experiments [30]. These conflicting reports about its demethylase activity have been put to bed with recent studies elucidating the role of JMJD6 as a demethylating enzyme in studies involving the regulation of transcriptional pause release [31], methylated estrogen receptors alpha (ER*α*) [32], RNA helicase A (RHA) in foot-mouth disease virus, etc. [33].

Today, JMJD6 is a well-recognized bifunctional enzyme with both demethylase and hydroxylase activities on a wide variety of target molecules presented in cytoplasm and the nucleus. JMJD6 is characterized as an RNA-interacting protein with lysyl hygroxylase activity validated in different cells using endogenous substrates; however, its possible role as arginine demethylase cannot be totally excluded, while the validation is still pending [34]. Its role in several important biological processes and association with a diverse set of pathological conditions attracted the interest of researchers across different fields of biology.

## 3. Structure of JMJD6

**Figure 1:**
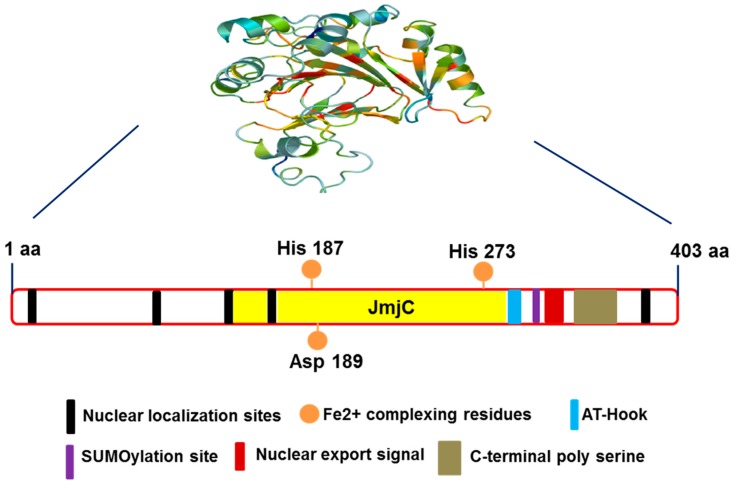
Predicted structure of Jumonji domain-containing protein 6 (JMJD6). AT-Hook: DNA-binding motif; JmjC: Jumonji C; SUMO: small ubiquitin-like modifier.

Structural analysis of JMJD6 protein revealed a Jumonji C (JMJC)-domain and other motifs, such as three nuclear localization signals (NLS), a DNA binding domain (AT-hook), a putative sumoylation site, and a polyserine domain (polyS) [30]. The core protein structural fold i.e., DSBH is surrounded by characteristic secondary structure elements. The structure of JMJD6 (summarized and designed by [35,36]) is presented in Figure 1. Crystallographic studies confirmed the structure of DSBH and also revealed iron-binding residues and residues involved in 2OG binding [37]. Hong et al., identified a positively charged area in a groove containing helix-turn-helix-like motif around the protein catalytic center that could possibly be responsible for JMJD6 interaction with DNA/RNA proteins. Electrophoretic mobility shift assays (EMSAs) using full-length JMJD6 proteins revealed efficient binding of JMJD6 to single stranded RNA (ssRNA) but not to double-stranded RNA (dsRNA), ssDNA, or dsDNA [38]. JMJD6 can exist in both monomeric and larger oligomeric forms. Also, JMJD6 can adopt an oligomeric form both in solution as well as cells [39,40]. By oligomerization, JMJD6 forms ring-like structures, and these structures form into fibrils upon deletion of the polyS sequence. The polyS also influences the sub-nuclear localization of JMJD6. In the presence of the polyS domain, JMJD6 was predominantly found in the nucleoplasm, whereas in the absence of polyS domain JMJD6 was found in the nucleolus and nuclear speckles [40].

## 4. Enzymatic Activity and Interactions of JMJD6

The two known enzymatic actions of JMJD6 include hydroxylation and demethylation. These reactions are dependent on the presence of cofactors such as Fe (II), 2OG and ascorbate. In the case of demethylation on arginine residues, the reaction proceeds by consuming 2OG to hydroxylate the methyl group, yielding an intermediary hemiaminal that is fragmented to yield formaldehyde and unmodified arginine residue [41,42,43]. Hydroxylation involves a sequential process, in which 2OG, oxygen and substrate, bind to the active site containing Fe (II). The resulting oxidative decarboxylation of 2OG generates an Fe (IV) oxo-species reactive with carbon–hydrogen bonds of substrates resulting in a hydroxylated product [44]. Initial experiments in human embryonic kidney (HEK) 293T cells to investigate the possible proteins interacting with JMJD6 led to identification of 40 such proteins. These included proteins related to messenger RNA (mRNA) processing and splicing, helicases, DNA binding proteins, DEAD box proteins (amino acid sequence D-E-A-D (asp-glu-ala-asp)), etc. [29].

## 5. Cancer and JMJD6

Multiple studies demonstrated the involvement of JMJD6 in cancer pathogenesis. Cancer is characterized by the unregulated proliferation and spread of abnormal cells. Apart from genetic mutations, epigenetic modifications can also disrupt gene expression to cause malignant cellular transformation [45]. As discovered, DNA methylation and histone modifications are two major epigenetic modifications. It is well established that global hypomethylation in many genomic sequences results in genomic instability and activation of proto-oncogenes, whereas site-specific hypermethylation of tumor suppressor genes results in loss of their protective functions, thus leading to cancer development and progression. Similarly, histone proteins are subjected to a wide variety of post-translational modifications—i.e., methylation, acetylation, ubiquitination, etc.—on specific residues that can lead to gene activation or repression depending on the type and site of modification [45]. The discovery of JMJD6 as an arginine demethylase on histone H3 at arginine 2 (H3R2) and histone H4 at arginine 3 (H4R3) led to the speculation about its role in influencing chromatin remodeling and gene expression [22]. Clinically, the association between aberrant expression of JMJD6 and aggressive cancer disease with poorer prognosis prompted further investigation to understand its role in tumorigenesis. Though alterations in the sequence of JMJD6 have not been identified in cancer, its overexpression is associated with various cancers, suggesting that it could constitute targets for therapeutic strategies [19]. We review the role of JMJD6 in selected cancers.

## 6. Oral Cancer and JMJD6

In the USA, cancer of the mouth and pharynx represents 3% of all new cancer cases, and more than 90% of all oral cancers belong to the subtype of oral squamous cell carcinoma (OSCC) [46]. Cancer stem cells (CSC) are defined as a small group of cancer cells responsible for initiation and maintenance of tumor growth by the processes of self-renewal and cell differentiation [47]. Earlier studies uncovered and validated the role of CSCs in OSCC [48]. CSCs are shown to play a vital role in various tumorigenic processes such as tumor progression, metastasis, and tumor recurrence, thereby leading to the sustenance of cancer and difficulty in formulating effective cancer treatment strategies [47].

Experiments conducted by Lee et al., in different OSCC cell lines, revealed that JMJD6 silencing inhibited self-renewal capacity, migration, and anchorage-independent growth ability [49]. On the other hand, the overexpression of JMJD6 in the same cell lines was associated with increased CSC migration, invasion, and resistance to cytotoxic drugs—including doxorubicin, methotrexate, and etoposide. These changes observed in the cell lines were the result of altered expression of various CSC-related genes, such as IL-4, Zeb1, Zeb2, Lin28A, and Lin28B by JMJD6. Among various cytokines involved in the regulation of CSC properties, interleukin 4 (IL-4) is known to inhibit apoptosis, enhance proliferation during metastasis, and confer resistance to chemotherapy drugs, thus promoting tumor formation and growth. Further studies revealed that JMJD6 lies upstream of IL-4 and regulates the expression of IL-4 by binding to its promoter region. This JMJD6 induced upregulation of IL-4 plays a crucial role as an inducer of CSC properties in OSCC.

In vitro studies showed higher levels of JMJD6 in OSCC cell lines than in non-malignant oral epithelial cell lines. These findings were further validated when in vivo studies showed higher levels of JMJD6 in OSCC when compared to normal tissues. Also, JMJD6 was consistently overexpressed in OSCC CSC populations compared to non-CSC populations [49]. All of these observations taken together support the hypothesis that JMJD6 is a novel molecular regulator of CSC phenotype in OSCC.

Though JMJD6 has been identified as an independent marker for poor prognosis in various cancers—such as lung adenocarcinoma, breast cancer, and colon cancer—its role in relation to OSCC is not absolutely clear. The recent identification of JMJD6–IL-4 axis as a novel regulator of oral CSCs is of particular interest given the plausibility that novel treatments targeting JMJD6 could be an effective treatment modality for oral cancer.

## 7. Colon Cancer and JMJD6

Colorectal cancer (CRC) is the third most common cancer diagnosed worldwide constituting about 10% of new cancer diagnoses [50]. Of the various intracellular signaling pathways implicated in CRC, the dysregulation of the p53 pathway is well accepted [51]. The p53 gene encoding for the p53 transcription factor plays a crucial role in regulation of cell division by inducing cell cycle arrest, apoptosis, or senescence through its downstream target genes depending upon the context to ensure cell survival and genomic stability [52]. Also, p53 gene mutations and a dysregulated p53 pathway in the context of CRC have been associated with more invasive and metastatic disease, poorer prognosis, resistance to chemotherapy, etc. [51].

In vitro and in vivo experiments using human colon carcinoma HCT116 cells not only showed a physical association between p53 and JMJD6, but also demonstrated a co-localization of p53 and JMJD6 within the nucleus. It was observed that JMJD6 hydroxylates p53 on lysine 382 (K382) of p53 in a 2OG and Fe (II)-dependent manner. It is postulated that this hydroxylation on K382 of p53 negatively regulates the p53 pathway by antagonizing the acetylation (enhancer of p53 transcription) of this site, thereby promoting the interaction of p53 with its negative regulator double minute X human homolog (MDMX) and downregulating transcriptional activity of p53. On the other hand, silencing JMJD6 increases p53 transcriptional activity, induces cell cycle arrest in the G1 phase, promotes cell apoptosis, and sensitizes cells to cytotoxic agents.

When studying the tumorigenic potential of JMJD6 in various human cancers (breast, lung, renal, pancreatic, liver, esophageal, colorectal, etc.), Wang et al., found the highest level of JMJD6 in colon cancer [53]. Further analysis in colon cancer samples revealed that the higher expression of JMJD6 positively correlated to depth of invasion, lymph node metastasis, advanced tumor node metastasis staging, and poor histologic grading of cancer. Survival rate analysis showed that patients with a high expression of JMJD6 lived for a significantly shorter duration than those with a lower expression of JMJD6. Based on immunohistochemistry findings in the normal tissue sections showing high expression of JMJD6 at the base of intestinal epithelium, it was hypothesized that JMJD6 is actively involved in the regulation of proliferation and differentiation of intestinal cells [53]. Though the exact mechanisms of JMJD6-mediated carcinogenesis are not yet fully understood, these observations lend support to the existing hypothesis implicating JMJD6 in the pathogenesis of various cancers [54,55]. The identification of JMJD6 as a novel biomarker for colon cancer prognosis and a target for novel therapeutic interventions warrants further study to elucidate the molecular mechanisms of its activity regulation and its biological functions.

## 8. Lung Cancer and JMJD6

Lung cancer is the most commonly diagnosed cancer worldwide. Based on histological findings, it can be classified into two broad categories, namely (i) Small-Cell Lung Carcinoma (SCLC); and (ii) Non-Small-Cell Lung Carcinoma (NSCLC) [56]. Adenocarcinoma of lung represents about 40% of all lung cancers [57]. It is not only common for current or former smokers, but also is the most common type of cancer seen in never-smokers [58].

Ji Zhang et al., studied the expression of JMJD6 mRNA and protein levels in archived lung adenocarcinoma tissue samples obtained from 154 patients who underwent surgical resection without prior radiotherapy or chemotherapy [59]. They used qRT-PCR (quantitative real-time polymerase reaction) and Western blot to characterize the relative JMJD6 mRNA and protein expression, respectively, and found that the JMJD6 mRNA level was significantly elevated in lung adenocarcinoma tissues compared with the corresponding non-tumorous lung tissues. Based on the percentage of JMJD6-positive tumor cells obtained by immunohistochemical staining, patients were subdivided into low expression group (*n* = 69) and high expression group (*n* = 85). When analyzed for clinical associations between lung adenocarcinoma and JMJD6 expression, the study group with high JMJD6 expression was positively associated with tumor size, pathological grade, and pleural invasion. Other studied variables—such as gender, age, and smoking history—had no significant relationship to JMJD6 protein expression. Kaplan–Meier analysis showed that lung adenocarcinoma patients with high JMJD6 expression had a significantly lower overall survival rate compared to those with low JMJD6 expression. Univariate and multivariate Cox regression studies revealed that JMJD6 expression was associated with decreased overall survival rate and was an independent prognostic factor for overall survival rate and adverse clinical outcome in patients with adenocarcinoma of the lung [59]. The mechanism for how JMJD6 expression regulates lung cancer severity is not clear. However, a possible explanation is related to the regulation of HOX genes (a subset of homeotic genes).

HOX genes are a subgroup of the homeobox family that encodes for Hox proteins. Hox proteins are transcription factors that can bind to specific nucleotide sequences on the DNA, resulting in either activation or repression of genes. These genes and encoded proteins play a crucial role in embryonic development. Also, the aberrant expression of these genes has been linked to both tumorigenesis and tumor suppression, depending on the context and the type of cancer [60]. In relation to lung cancer, previous studies have shown that the upregulation of HOXB9 (Homeobox protein Hox-B9) is associated with lower patient survival and is a predictor of poorer clinical outcomes [61]. When studying the post translational modifications of HOXB9, Wan et al., were able to demonstrate in vitro and in vivo acetylation. Acetylation of HOXB9 is a dynamic process, in which acetyltransferase PCAF (P300/CBP-associated factor) acetylates and deacetylase sirtuin 1 (SIRT1) deacetylates. A specific site, K27 is a main acetylation site of HOXB9. The acetylation at K27 of HOXB9 inhibits the function of HOXB9 in promoting lung cancer cell migration and tumor growth. Interestingly, based on chromatin immunoprecipitation (ChIP) assays, JMJD6 is a direct downstream target of HOXB9. The acetylation deficient mutant of HOXB9 K27R showed increased binding ability to the promoter region of JMJD6 when compared to that of the wild type and resulted in upregulation of JMJD6 at both mRNA and protein levels. In vivo studies in a cohort of 75 lung adenocarcinoma patients showed that people with higher levels of HOXB9 acetylation had significantly better overall survival, smaller tumor size, and lower lymph node metastasis when compared to those with lower levels of HOXB9 acetylation [62].

## 9. Breast Cancer and JMJD6

In addition to cancer of other organs, there is a link between the disease progression and JMJD6 expression in breast cancer cells. As shown by [54], JMJD6 expression in tumors was associated with worse outcomes. Furthermore, loss of JMJD6 consistently resulted in suppressed proliferation, but not apoptosis, while overexpression promoted cell growth. The authors observed the inverse relation of JMJD6 and transforming growth factor-beta 2 (TGF-*β*2) expression. Some studies claimed the link between JMJD6 and ER*α* arginine methylation, indicating ER*α* as a new substrate for JMJD6. This shows that JMJD6 demethylase activity is a regulator of rapid physiological responses to estrogen [32]. The studies from the same group confirmed that JMJD6 is a marker of poor prognosis in breast cancer [63]. Another breast cancer-related study demonstrated the role of JMJD6 amplification in tumor cells as cooperating with c-Myc, which leads to reduced p53 levels, tumor metastases, progression, and increased cellular transformation [64]. Thus, in breast cancer, JMJD6 serves as a driver of cell proliferation and motility and an indicator of poor prognosis.

## 10. JMJD6 and Viruses

The clinically important correlations between JMJD6 expression and severity/outcomes are not only the case for oncological diseases, but are also observed in some virus-related situations. Interestingly, in chronic hepatitis B (CHB), JMJD6 has been shown to promote cluster of differentiation 4 (CD4+) T-cell proliferation by suppressing cyclin-dependent kinase inhibitor 3 (CDKN3) mRNA expression (which is known to inhibit cell cycle progression by binding to CDK2 kinase) [65].

Another example of JMJD6-virus interactions is a foot-and-mouth disease virus (FMDV), a member of the *Aphthovirus* genus of the *Picornaviridae* family is a positive-sense single stranded RNA virus which is highly infectious pathogen of cloven-hoofed animals, including livestock such as pigs and cattle [66]. FMDV attaches to and enters the host cell by receptor-mediated endocytosis. The known receptors for attachment of the virus include integrin heterodimers and an alternative receptor heparan sulphate (HS). Of late, a third receptor pathway has been identified when studies showed evidence of the ability of mutant viruses derived from serotype C FMDV and A-SIR #42 to infect the CHO derived cell line (CHO 677), lacking both the integrin and HS receptors. Later it was identified that these mutant varieties of the virus possessed a unique set of amino acid substitutions (E95K/96L) in the Virus Pathogen Resource (VP1) protein which were proposed to be responsible for third receptor tropism. Further analysis revealed that the presence of JMJD6 is required for the entry of virus through the third receptor pathway. To better understand the role of JMJD6 in the host cell entry of FMDV, Lawrence et al., conducted JMJD6 knockdown experiments using JMJD6-specific small interfering RNAs (siRNAs) and the relative virus titers were measured by plaque assay [66]. They found about a 65% reduction in the viral titers, and this finding was attributed to the observed partial reduction in cytoplasmic/cell surface JMJD6 content following siRNA treatment. The inhibitors of the classical complement dependent pathway (CCP; hypertonic sucrose) and the caveolae-dependent pathway (nystatin) were utilized to understand the internalization route of the JMJD6-dependent alternative receptor pathway. Western blot analysis of the virus-infected cells treated with inhibitors revealed that increasing the concentration of hypertonic sucrose resulted in reduced detection of viral proteins, whereas increasing the concentration of nystatin had no effect. Further, 0.4 M sucrose was identified to be sufficient to impede the infection by JMJD6-FMDV. Immunofluorescent microscopy used to study the movement of JMJD6 from cell membrane to nucleus after infection with JMJD6-FMDV showed failure of internalization of JMJD6 by the mutant virus in the presence of hypertonic sucrose, while nystatin treatment did not impede the JMJD6 internalization. These findings suggest that JMJD6-FDMV endocytosis is mediated by CCPs.

Furthermore, in vivo studies conducted by Lawrence et al., to understand the virulence and infection dynamics of mutant JMJD6-FMDV, revealed that animals infected by a simulated-natural (aerosol) route when compared to the less natural intraepithelial lingual (IEL) route showed no clinical signs of infection (vesicles, ameness, fever) and no viremia at any time point. On the other hand, IEL-inoculated animals had a clinical course of the disease that was indistinguishable from the virulent parental FMDV A24-WT. Sequencing of the viral particles isolated from vesicular fluid and tissue samples of these animals confirmed no alterations in the capsid sequence, showing that the JMJD6-FMDV mutant virus was indeed causing the clinical disease. The authors also postulate that differences in the degree of virulence based on the route of inoculation could be due to the non-accessibility of the key components of the third receptor pathway by aerosol route of inoculation [66,67].

The link between JMJD6 expression and the resistance to viruses may be also based on the ability of JMJD6 to interfere with host innate immunity [68]. The crucial components of the innate immune system are toll-like receptors (TLRs), which are membrane spanning proteins expressed in both immune cells (macrophages, natural killer (NK) cells, dendritic cells, lymphocytes) and non-immune cells (epithelial cells, endothelial cells, fibroblasts, etc.) [69]. Their main function is to recognize specific antigen patterns of pathogens and activate immune responses through various cell signaling pathways [70]. A subset of TLRs—namely, TLR3, TLR7/8, and TLR9—are widely implicated in recognition of viral nucleic acid sequences and activation of either myeloid differentiation primary response gene 88 (MYD88)-dependent or TIR-domain-containing adapter-inducing interferon-β (TRIF)-dependent pathway to induce expression of inflammatory cytokines and initiate adaptive immune response [71]. An important mediator in these TLR pathways is TNF receptor-associated factor 6 (TRAF6), an E3 ubiquitin ligase whose activity depends on its methylation status. Tikhanovich et al. demonstrated that TRAF6 is demethylated by JMJD6, resulting in its activation and upregulation of downstream Nuclear Factor kappa-light-chain-enhancer of activated B cells (NF-𝜅B) pathways [72]. Two TRAF6 arginine residues, namely Arg-88 and Arg-125, have been identified to play a key role in its regulation. Substitutions or mutations at these residues were resulted in impaired interactions of TRAF6 with JMJD6 and PRTM1. In vitro and in vivo studies in both hepatocytes and macrophages demonstrated that TRAF6 indeed is a target for JMJD6, and is activated upon demethylation by JMJD6. Overexpression and knockdown experiments identified a direct relationship between JMJD6 levels and TRAF6 ubiquitin ligase activity. It was concluded that the basal activity of the TLR pathway is determined by the methylation balance of TRAF6 maintained by protein methyltransferase 1 (PRMT1) and JMJD6. In a normal state, a high PRMT1/JMJD6 ratio was associated with inactive TRAF6 and inactive TLR signaling pathways, thus preventing inappropriate inflammation. On ligand exposure, transient degradation of PRMT1 shifted the equilibrium towards demethylation of TRAF6 by JMJD6, resulting in maximal activation of downstream signaling to generate appropriate immune responses. When an immune response was no longer needed, the restoration of PRMT1 levels aborted the signaling process. Though the exact mechanisms that bring about these changes are not yet understood, this novel discovery remains a step in the right direction towards the understanding of dynamic protein arginine methylation and other factors regulating innate immunity [72].

Another integral part of the innate immunity is interferons (IFNs) type 1, cytokines released by host cells in response to diverse antigenic stimuli. They not only exhibit antiviral activity by increasing the expression of antiviral genes, but also by activating other immune cells, promoting apoptosis and antigen presentation via upregulation of immunoproteasome and major histocompatibility complex (MHC) molecules, etc. [73]. In viral infections, one of the most potent innate immunity factors is IFNα, a major subtype of IFNs. IFNα activates the Janus Kinase/Signal Transducer and Activator of Transcription (JAK-STAT) pathway to induce the formation of a transcription complex, called the IFN-stimulated gene factor-3 (ISGF3) complex, that translocates to the nucleus to bind to specific nucleotide sequences called IFN-stimulated response elements (ISREs) in the promoter regions of genes identified as IFN stimulated genes (ISGs) [74]. One of the crucial determinants of activation of antiviral genes is the methylation status of STAT1 molecules as methylated STAT1 is needed to bind to DNA. Methylation of STAT1 is regulated by PRMT1, which methylates this protein on arginine residues [75]. Recently, we have shown that STAT1 methylation was impaired by exposure to ethanol metabolite, acetaldehyde, and Hepatitis C virus (HCV) [76,77].

**Figure 2:**
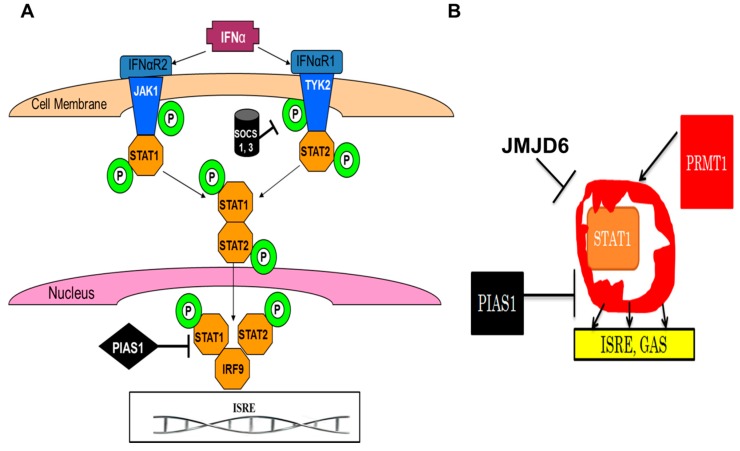
Interferon (IFN) type 1 signaling; regulation by methylation. (**A**) IFNα signaling via the Janus Kinase/Signal Transducer and Activator of Transcription (JAK-STAT1) pathway; (**B**) Regulation of STAT1 methylation by protein methyltransferase 1 (PRMT1) and JMJD6 (hypothesis). Demethylation of STAT1 by JMJD6 suppresses IFN type 1 signaling and interferon-stimulated gene activation, thereby promoting the spread of infection (as an example, this may happen in Hepatitis C virus-infected hepatocytes exposed to alcohol). IFNαR: interferon receptors alpha; ISRE: interferon-sensitive response element; GAS: gamma-interferon activated sequence; JAK1 and TYK2: Janus kinases; P: phosphorylation; PIAS1: protein inhibitor of activated STAT1; SOCS: suppressors of cytokine signaling.

However, in addition to decreased PRMT1 activity, the methylation status of STAT1 may also depend on demethylase JMJD6, which removes methyl groups from arginine residue. When studying the effects of alcohol metabolites (acetaldehyde) and HCV infection on expression of JMJD6 in hepatocytes, we have demonstrated that both acetaldehyde and HCV increase JMJD6 expression at the levels of both mRNA and protein. Overexpression and silencing of JMJD6 showed a direct relationship between JMJD6 and HCV RNA levels. Concurrently, the upregulation of JMJD6 was associated with suppressed methylation of STAT1 and decreased activation of ISGs (briefly described in [78]). We thus hypothesize that JMJD6-induced demethylation of STAT1 suppresses activation of antiviral genes by attenuating JAK-STAT signaling, resulting in enhanced HCV replication, and this effect of JMJD6 is further exacerbated by exposure to alcohol [79]. Figure 2 shows the involvement of JMJD6 in the suppression of IFN*α* signaling through the JAK-STAT1 pathway in viral infections. In addition to viruses, in the studies from our laboratory, the alcohol metabolite, acetaldehyde, significantly induced the expression of JMJD6 mRNA in Huh 7.5 cells and primary human hepatocytes even in the absence of virus. Interestingly, in our hands, JMJD6 demethylated STAT1 only on arginine, but not on lysine residues, while previously reported effects of acetaldehyde were broader than that since it suppressed both arginine and lysine STAT1 methylation [76]. This corresponds to the observations by Hahn et al., that shuttling in and out of the nucleus, JMJD6 also does not demethylate histones (H3K4, H3K9, H3K27, H3K36, and H4K20) on lysine residues [80].

In conclusion, the bifunctional enzyme JMJD6 plays an important, but underappreciated, role in the regulation of histone/protein methylation, thereby amplifying pathogenesis of many diseases, including cancers, viral hepatitis, alcoholic liver disease, etc. More studies are necessary to identify the clinical situations negatively affected by JMJD6 overexpression and to elucidate the mechanisms by which JMJD6 contributes to the regulation of disease severity and outcomes. The impact of JMJD6 on chronic injury progression makes this enzyme an attractive target for therapeutic interventions.
